# The Effect of Environmental Policy Uncertainty on Enterprises’ Pollution Emissions: Evidence from Chinese Industrial Enterprise

**DOI:** 10.3390/ijerph19169849

**Published:** 2022-08-10

**Authors:** Dong Le, Fei Ren, Yiding Tang, Yuke Zhu

**Affiliations:** 1 Economics School, Zhongnan University of Economics and Law, Nanhu Avenue 182, Wuhan 430073, China; 2 School of Economics and Trade, Hunan University, Fenglin Road, Changsha 410006, China

**Keywords:** environmental policy uncertainty, enterprise pollution emission, enterprise innovation, human capital, the foreign investment

## Abstract

In response to the global call for emission reduction, China has assumed international responsibility for energy conservation and emission reduction by enacting several environmental policies to save energy and reduce consumption. However, it is debatable whether the increased uncertainty in environmental policies negatively affects firms’ emission reduction. Few studies have examined this relationship based on micro-level data. Therefore, this study constructs a theoretical framework of environmental policy uncertainty affecting firms’ pollution emissions. Based on comprehensive data from the Chinese Industrial Enterprise Database, the Chinese Industrial Enterprise Pollution Emission Database, and the Chinese Patent Database from 2002 to 2014, we empirically analyzed the impact of environmental policy uncertainty on firms’ pollution emissions. The results show that (1) environmental policy uncertainty significantly aggravates the pollution emission intensity of industrial enterprises; (2) environmental policy uncertainty inhibits the improvement of enterprises’ innovation capacity, reduces their human capital stock and foreign investment, and aggravates their pollution emission; (3) environmental policy uncertainty has significant heterogeneity on enterprise pollution emissions, that is, environmental policy uncertainty has a greater impact on non-export enterprises, large enterprises, young enterprises, capital-intensive enterprises, state-owned enterprises, and enterprises in polluting industries and central regions. This study provides a useful reference for the improvement of environmental policy and the green transformation of enterprises.

## 1. Introduction

Approximately four decades of economic growth made China the world’s second-largest economy in 2010. However, this economic development brought in its wake serious resource constraints, high levels of pollution by enterprises, and the degradation of the ecosystem. According to the 2020 Report on China’s Ecological and Environmental Conditions, only 59.9% of cities across the country have acceptable air standards, high pollution emissions cause 1.2–1.4 million deaths annually [1], and direct and indirect economic losses are pegged at 1–8% of China’s GDP [2]. As the Central Committee of the Communist Party of China has proposed to strengthen socialist ecological civilization construction, the government has issued a series of rules and regulations to promote enterprise emission reduction, including the Action Plan for Air Pollution Prevention and Control (2013) [3], the Implementation Regulations of the Environmental Protection Tax Law of the People’s Republic of China (2017), and the Guiding Opinions on Building a Modern Environmental Governance System (2020). Government regulations are an important measure to control the pollution discharge of enterprises. Given that the market is a dynamic process, environmental policies need to be dynamically adjusted in real time according to factors such as economic development and technological progress. These policies have, no doubt, effectively reduced the contradiction between economic development and enterprise emission reduction but have also increased the uncertainty of environmental policies. Research suggests that environmental policy uncertainty itself may lead to economic decline [4]. Currently, China’s economy is in the transition phase, and environmental policy uncertainty is an important feature of this period. However, few studies have focused on whether an increase in environmental policy uncertainty impacts enterprise emission reduction. It is rarely mentioned in the existing literature. In the context of the green transformation development of enterprises, research on environmental policy uncertainty and enterprise emission reduction has immense theoretical and practical significance.

### 1.1. Literature Review

In the context of the green transformation and development of enterprises, enterprise pollution emission has become a hot topic in academia. Additionally, government regulation policy has an immense impact on enterprise pollution emission. If environmental policies are stringent or frequent, the technological progress of enterprises will be hindered, which will eventually lead to the development of enterprises [5]. Burtraw et al. [6] found that government energy-saving and consumption-reduction policies significantly promote enterprise emission reduction; additionally, they calculated the economic benefits generated by enterprise emission reduction. Oueslati [7] proposed that carbon emission trading rights and carbon taxes can significantly reduce carbon emissions; additionally, carbon emission trading rights can promote the improvement of low-carbon technologies of enterprises and enhance their market competitiveness [8].

#### 1.1.1. Influencing Factors of Enterprise Pollution Discharge

Regarding the influencing factors of enterprise pollution discharge, the existing literature mainly focuses on technological innovation, human capital, and foreign investment. The development experience of developed countries shows that technological innovation can fundamentally solve the problem of excessive pollution emissions of enterprises and improve the competitiveness of enterprises’ products, and the improvement of green innovation ability can help enterprises occupy market share [9]. When the intensity of environmental regulation increases, the innovation potential of enterprises will be stimulated to realize the coordinated development of environmental protection and the green development of enterprises [10]. Popp [11] proposed that government innovation support policies and enterprise innovation behavior can form a complementary situation. Government innovation support policies can motivate enterprises toward green production and improve enterprises’ pollution discharge technology, in addition to promoting the reduction in enterprises’ pollution emission intensity. Additionally, Porter [12] suggested that companies seek new technologies to reduce emissions and pollution costs.

Human capital—an important aspect of economic efficiency and green development—influences the maximization of resource utilization efficiency and breaking through the constraint of diminishing marginal returns. Investing in enterprise human capital enhances the quality of managers and laborers, thereby improving the energy utilization efficiency of enterprises. When enterprise human capital stock is high, it can significantly improve the absorption capacity and application ability of energy-saving technologies [13]. Kurtz et al. [14] used sample data from different countries to find that human capital can significantly improve the energy utilization efficiency of enterprises and reduce the unintended output in the production process. Lan et al. [15] identified that regional enterprises with low human capital are more prone to the “pollution paradise hypothesis.” Lan and Munro [3] found that human capital transcends the constraint of diminishing marginal returns through positive externalities and reduces the intensity of enterprise pollution emission.

The influence of foreign investment on enterprises’ pollution emissions has not been determined yet, and research has mainly focused on the “pollution paradise hypothesis” and “pollution halo hypothesis.” The pollution paradise hypothesis holds that foreign-funded enterprises tend to move to countries with weak environmental regulations. Here, local enterprises engage in a vicious competition with transferring enterprises—most of which are pollution-intensive industries—reducing investment in green technology to save costs and increasing pollution emission intensity [16]. The pollution halo hypothesis holds that foreign investment reduces enterprise pollution emission intensity. On the one hand, when industrial transfer occurs in developed countries, foreign-funded enterprises often have more advanced pollution discharge technologies and better management systems. The competition encourages local enterprises to improve their own production technologies and management systems through imitation [17]. On the other hand, the products of foreign-funded enterprises are more environmentally friendly, and these enterprises require local suppliers to adopt green and efficient production technologies to guarantee their product advantage. At this time, foreign enterprises will provide advanced green production technology and a perfect management mode to local suppliers, so as to strengthen the spillover effect of advanced technology and reduce enterprise pollution emissions [18].

#### 1.1.2. Discussion about Environmental Policy Uncertainty

Existing studies on environmental policy uncertainty focus on its connotations and impacts on enterprise investment, technological innovation, and economic development. Uncertainty implies that actors make decisions without access to complete information, and therefore, the specific impact of their decisions cannot be accurately predicted. When the object of uncertainty is environmental policy, then environmental policy uncertainty is formed [19]. Environmental policy uncertainty refers to the inability of economic entities to accurately predict when, how, and whether the government will change the current environmental policy. Since the central government proposed the comprehensive protection of the ecology and environment, local governments have frequently adjusted environmental regulations to reduce enterprise emission; so, the uncertainty of environmental policy has become the main feature of the economic transition period [20]. Environmental policy uncertainty is not a specific policy, but a measure of how often environmental policy changes. The greater the value of environmental policy uncertainty, the more frequent the government’s environmental policy changes. The uncertainty of environmental policy has a negative impact on foreign investment, employment, and enterprise innovation. When the environment shows a dynamic trend of change, it is difficult for enterprises to predict future results, which increases the cost of enterprise decision making [21]. With the deepening of China’s economic transformation, the influx of foreign capital, and stringent environmental regulations, Chinese enterprises are facing increasing uncertainty [22]. Increased environmental policy uncertainty raises financial risks and incentivizes risk avoidance, which pushes enterprises to reduce investment in pollution reduction and aggravates pollution emissions [23].

#### 1.1.3. Summary

To date, studies have been conducted on enterprises’ pollution emissions from the perspectives of government regulation policies, influencing factors, the connotations of environmental policy uncertainty, and its influence on foreign investment, employment, and enterprise innovation. However, little research has been performed on whether increasing environmental policy uncertainty impacts enterprise pollution emissions. Additionally, due to the lack of an effective micro-basis of macro-level research, the emission reduction path of the macro-level influence mechanism acting on micro-enterprises is not clear. In fact, how to deal with the increasing uncertainty of environmental policy is the basis and key to understand the relationship between policy fluctuation and emission reduction at the macro level. Therefore, based on the combined data of the China Industrial Enterprise Database, China Industrial Enterprise Pollution Emission Database, and China Patent Database from 2002 to 2014, this study illustrates the impact and mechanism of environmental policy uncertainty on enterprise pollution emissions from the perspective of micro-enterprises.

## 2. Materials and Methods

### 2.1. Theoretical Model

#### 2.1.1. Environmental Policy Uncertainty Worsens Enterprise Pollution Emissions by Inhibiting Enterprise Innovation

Based on the research by Bloom, Bond, and Van Reenen [24], this study expounds how the uncertainty of environmental policy affects the pollution emission of enterprises. It is assumed that the production of enterprises in period t conforms to the Cobb–Douglas production function, and the specific function form is as follows:

(1) $$F=A_{t}K_{t}^{\alpha }L_{t}^{\beta }G_{t}^{1-\alpha -\beta }$$

In Formula (1), $A_{t}$ refers to the production conditions of the enterprise, mainly including its own production conditions and external factors; $K_{t}$ is the capital stock of the enterprise; $L_{t}$ is the labor stock of the enterprise; and $G_{t}$ is the knowledge stock of the enterprise. It is assumed that $G_{t}$ is proportional to the enterprise’s innovation capability μ, so:

(2) $$G_{t}=a\mu +\varepsilon$$

At this point, the demand function faced by the enterprise is:

(3) $$D_{t}=B_{t}P^{-\frac{1}{\gamma }}$$

where *B* is the demand shock faced by the enterprise. Based on the relationship between demand and supply, the income function of the enterprise is as follows:

(4) $$\begin{array}{ll} M(A_{t},B_{t},K_{t},L_{t},G_{t}) & ={\left(A_{t},K_{t}^{\alpha },L_{t}^{\beta },G_{t}^{1-\alpha -\beta }\right)}^{1-\gamma }B_{t}^{\lambda }\\ & =A_{t}^{1-\gamma }B_{t}^{\gamma }K_{t}^{\alpha (1-\lambda )}L_{t}^{\beta (1-\gamma )}G_{t}^{\left(1-\alpha -\beta )(1-\gamma \right)} \end{array}$$

We defined $X_{t}^{\varphi }=Z_{t}^{1-\gamma }B_{t}^{\gamma }$, where *X* indicates that the production and investment of enterprises are affected by the uncertainty of environmental policies, making $X_{t}^{\varphi }=f(\lambda )={\left(b\lambda +n\right)}^{\varphi },\;b>0$. To judge the relationship between environmental policy uncertainty λ and innovation capability μ, we further simplified the model along with the research ideas of Bloom, Bond, and Van Reenen [24], assuming that capital *K* and labor *L* are completely flexible and their variable costs are 0. We substituted Formula (2) into Formula (4), and the return function is:

(5) $$M(\lambda ,\mu )=K(b\lambda +n{)}^{\varphi }{\left(a\mu +m\right)}^{1-\varphi }$$

To a homogeneous equation:

(6) M=Kφ(bλ+n)+(1−φ)(aμ+m)

The emission reduction in enterprise pollution is not only affected by the original pollutant discharge $Q^{*}$ but is also closely related to the enterprise innovation ability. There is a positive correlation between enterprise innovation capacity and emission reduction. *S* is the influence coefficient of enterprise innovation capacity on enterprise emission reduction (0 < *s* < 1). Additionally, the technological innovation of enterprises reduces the cost of enterprises’ pollution emission; e is the proportion of the impact of technological innovation on the cost of enterprises’ emission reduction (0 < *e* < 1). Additionally, the innovation cost of enterprises is affected by the uncertainty of environmental policy and innovation capability.

Assume that the total cost of the enterprise is C, which mainly includes emission cost $C_{1}$ and innovation cost $C_{2}$, so:

(7) $$C_{1}=e\mu \left(Q^{*}(1-s\mu )\right)$$

(8) $$C_{2}=\lambda \delta \mu$$

In Formula (8), δ measures the impact of environmental policy uncertainty on innovation capability (δ<0). Then, the total cost is:

(9) $$C=C_{1}+C_{2}$$

(10) $$C=e\mu \left(Q^{*}(1-s\mu )\right)+\lambda \delta \mu$$

The total profit function of the enterprise is:

(11) $$R=K\varphi (b\lambda +n)+(1-\varphi )(a\mu +m)-e\mu \left(Q^{*}(1-s\mu )\right)-\lambda \delta \mu$$

When profit is maximized, the first derivative of profit is 0, then:

(12) $$\frac{\partial R}{\partial \mu^{*}}=a(1-\varphi )-eQ^{*}+2eQ^{*}s\mu^{*}-\delta \lambda =0$$

According to Formula (12), the relationship between environmental policy uncertainty λ and enterprise innovation μ is as follows:

(13) $$\frac{\partial \lambda }{\partial \mu^{*}}=\frac{2esQ^{*}}{\delta }<0$$

According to Formula (13), the uncertainty of environmental policy will inhibit the improvement of enterprises’ innovation ability, whereas the technological innovation of enterprises will promote the upgrading of industrial structure, improve energy utilization efficiency, and further reduce enterprises’ pollution emissions [25,26]. Therefore, environmental policy uncertainty can inhibit enterprise innovation and aggravate enterprise pollution emissions.

#### 2.1.2. Environmental Policy Uncertainty Increases Enterprise Pollution Emissions by Inhibiting the Increase in Human Capital Stock

The production function is adjusted on the basis of Formula (1), and the meaning of the letters remains unchanged. The specific function form is as follows:

(14) $$F=A_{t}K_{t}{}^{\alpha }G_{t}^{\beta }L_{t}^{1-\alpha -\beta }$$

The larger the inflow of labor, the larger the stock of human capital [27]. The specific function form is as follows:

(15) $$L_{t}=cl+\xi$$

There is a positive correlation between the stock of human capital and emission reduction, and *s*_1_ is the influence coefficient of the stock of human capital on emission reduction (0 < *s*_1_ < 1). Human capital can internalize technical knowledge to overcome the diminishing marginal returns of production and reduce the cost of enterprise pollution emission. *e*_1_ is the proportion of human capital affecting enterprise emission reduction cost (0 < *e*_1_ < 1). The cost of introducing human capital is affected by environmental policy uncertainty and the effect of human capital promoting emission reduction.

Assume that the total cost of the enterprise is C, which mainly includes emission cost $C_{1}$ and human capital introduction cost $C_{2}$, so:

(16) $$C_{1}=e_{1}l\left(Q^{*}(1-s_{1}l)\right)$$

(17) $$C_{2}=\lambda \delta_{1}l$$

$\delta_{1}$ is to measure the impact of environmental policy uncertainty on the cost of introducing human capital ($\delta_{1}<0$). Through the derivation of Equations (9)–(12), it can be concluded that:

(18) $$\frac{\partial \lambda }{\partial l^{*}}=\frac{{2e}_{1}s_{1}Q^{*}}{\delta_{1}}<0$$

According to Formula (18), the uncertainty of environmental policy will inhibit the improvement of enterprise human capital stock. The new economic growth theory states that human capital can overcome the law of diminishing marginal returns on means of production and then realize economic growth and enterprise emission reduction [28,29]. Therefore, environmental policy uncertainty can aggravate enterprises’ pollution emission by inhibiting the increase in enterprises’ human capital.

#### 2.1.3. Environmental Policy Uncertainty Worsens Pollution Emissions by Inhibiting Foreign Investment

The production function is adjusted on the basis of Formula (1), and the meaning of the letters remains unchanged. The specific function form is as follows:

(19) $$F=A_{t}G_{t}^{\alpha }L_{t}^{\beta }K_{t}{}^{1-\alpha -\beta }$$

As foreign investment will increase the production and working capital of enterprises, it is assumed that $K_{t}$ is proportional to the foreign investment of enterprises, and the specific function form is as follows:

(20) $$K_{t}=df+\zeta$$

The increase in foreign investment makes enterprises strict in the formulation of environmental standards, and most multinational companies will implement international environmental standards, thus reducing the pollution emissions of enterprises. The amount of foreign investment is positively correlated with emission reduction, and *s*_2_ is the influence coefficient of foreign investment on emission reduction (0 < *s*_2_ < 1). Foreign investment can expand the production scale of enterprises to produce scale effect and reduce the pollution emission cost of enterprises. *e*_2_ (0 < *e*_2_ < 1) is the proportion of foreign investment affecting enterprise emission reduction cost, which is affected by both environmental policy uncertainty and foreign investment income.

Assume that the total cost of the enterprise is C, which mainly includes emission cost $C_{1}$ and human capital introduction cost $C_{2}$, so:

(21) $$C_{1}=e_{2}f\left(Q^{*}(1-s_{2}f)\right)$$

(22) $$C_{2}=\lambda \delta_{2}f$$

$\delta_{2}$$\left(\delta_{2}<0\right)$ is to measure the impact of environmental policy uncertainty on foreign investment returns. Through the derivation of Equations (9)–(12), it can be concluded that:

(23) $$\frac{\partial \lambda }{\partial f^{*}}=\frac{2e_{2}s_{2}Q^{*}}{\delta_{2}}<0$$

Increased foreign investment can significantly improve financial support for enterprises to reduce emissions. Additionally, foreign enterprises bring advanced emission reduction technologies that facilitate the implementation of international environmental standards in China. Moreover, by introducing green cleaning products from the home country, foreign investment has a clean technology spillover effect on its upstream and downstream industries, which is conducive to promoting emission reduction in domestic enterprises. Therefore, environmental policy uncertainty can inhibit foreign investment and aggravate the pollution emission of enterprises.

### 2.2. Mechanism Analysis

Currently, China’s economy is in a transition phase, and the government’s policy environment is experiencing significant fluctuations. The main feature of this phase is the increasing fluctuations and uncertainty of environmental policy. On the one hand, advanced production and pollution discharge technologies require enormous human and material investment, whereas the positive externality of technological innovation reduces the willingness of enterprises to invest in innovation. Additionally, environmental policy uncertainty intensifies the severe fluctuation of micro-enterprise performance, which increases the difficulty of operation. Enterprise managers will avoid the uncertainty of investment returns as much as possible because of their risk-averse and profit-oriented mindset and pay more attention to short-term investment and investment with definite returns. Then, they will reduce the investment in R&D [30]. Additionally, environmental policy uncertainty reduces the willingness of enterprise managers to invest in innovation, which lowers enterprise innovation ability [31]. On the other hand, the impact of such uncertainty on the production and earnings of enterprises is unknown. Enterprises’ earnings will also fluctuate because of the increased uncertainty of environmental policies. When the fluctuation of enterprises’ earnings is high, information asymmetry between enterprises and external investors increases accordingly, and creditors have difficulty predicting the likelihood of recovery and may reduce their risk by raising loan rates, demanding guarantees, or even not lending. Additionally, existing shareholders want to receive more cash dividends in the case of unexpected circumstances caused by uncertainty, and potential investors demand a higher risk premium, which leads to higher equity financing costs. Therefore, the increased uncertainty of environmental policies increases the financing difficulty of enterprises, forcing them to retain abundant cash to cope with the impact of environmental policy uncertainties. The lack of funds reduces enterprises’ innovation investment willingness. Therefore, an increase in environmental policy uncertainty inhibits the improvement of enterprise innovation ability. Advanced green production technology can improve the energy efficiency of enterprises, help enterprises break the constraints of resources and environment, and promote the reduction in enterprise emissions. In summary, this study proposes Hypothesis 1.

**Hypothesis** **1** **(H1).** *Environmental policy uncertainty can inhibit the improvement of enterprises’ innovation ability and aggravate enterprises’ pollution emission.*

When the uncertainty of environmental policy increases, it induces enterprise managers to make investments with definite short-term benefits. The tendency of enterprises to choose investment projects with higher risks and less obvious short-term returns will be weakened. The human capital investment of enterprises has the characteristic of not having an obvious income effect, and relative to other factors of production, human capital adjustment cost is low [32]. In order to cope with the unknown situation brought by the uncertainty of environmental policies, enterprises are more inclined to keep more cash to withstand the unknown risks they may face, and enterprises will reduce the investment in human capital. Human capital input is mainly divided into employee training and employee health input. When enterprises reduce employee training input, it is difficult for employees to improve their working skills. At the same time, in order to reduce expenses, enterprises will also increase the labor supply time of employees. Employees are also faced with the risk of being fired and income reduction, which damages the physical and mental health of employees. As a result, the healthy human capital of employees is reduced. When the health of employees is damaged, their enthusiasm to participate in labor will also be reduced. Additionally, the more unstable the enterprise environment, the stronger the mobility of employees, which also makes the implementation of internal control more difficult. It is difficult to maintain the stock of human capital effectively and stably. As an important aspect of economic and efficient green development, human capital plays a significant role in breaking through the constraint of diminishing marginal returns and maximizes resource utilization efficiency. On the one hand, human capital can reduce the increase in pollution emissions caused by low-level labor. A high level of human capital can guide the transformation of a low-skilled labor force to a high-skilled labor force and optimize the human capital structure of enterprises [33]. On the other hand, it promotes technological innovation for emission reduction and gives full play to the energy-saving production advantages of products, thus maximizing resource utilization efficiency and reducing the enterprise pollution emission intensity [34]. In summary, this study proposes Hypothesis 2.

**Hypothesis** **2** **(H2).** *The uncertainty of environmental policy increases the pollution emission of enterprises by inhibiting the increase in human capital stock of enterprises.*

The fundamental purpose of foreign investments is profit. When the uncertainty of environmental policy increases, it becomes difficult to accurately predict the income of foreign investment. To maximize interests and avoid risks, investors tend to invest in enterprises with clear and stable returns; they may even reduce investments in domestic companies because it is difficult to accurately control the risk of investment returns. The uncertainty of environmental policies has aggravated the fluctuation of enterprise performance and raised the financing difficulty of enterprises, which has hindered the production and operation of domestic enterprises. In the case of uncertain returns, foreign enterprises further reduce investment to reduce risks, so the increase in environmental policy uncertainty reduces foreign investment. Foreign investment has been a key component of economic growth and an important means of transferring modern technology and providing employment to host countries. Especially in recent years, inflows of foreign investment have become more important than international trade [35]. Foreign investment can not only increase the formation of resources and capital, but more importantly, it is an important channel for transferring production technology, entering new international markets, improving production capacity, and reducing unemployment—these are even more important for developing countries. Increased foreign investment has made companies more stringent in setting environmental standards. Most multinational companies implement international environmental standards, which reduce their emissions. Moreover, foreign investment can expand the production scale of enterprises to produce a scale effect and reduce the cost of pollution emission. Therefore, with the increase in foreign investment resulting in the spillover of green production technology, the enterprise pollution emission intensity is reduced [36]. In conclusion, this study proposes Hypothesis 3.

**Hypothesis** **3** **(H3).** *Environmental policy uncertainty worsens pollution emissions by inhibiting foreign investment.*

### 2.3. Models, Variables, and Data

#### 2.3.1. Research Model 

To verify the impact of environmental policy uncertainty on enterprise productivity, the benchmark model is set as follows:

(24) $$ei_{ijkt}=\beta_{0}+\beta_{1}epu_{ijkt}+\beta_{2}X_{ijkt}+\lambda_{i}+\lambda_{j}+\lambda_{k}+\lambda_{t}+\varepsilon_{ijkt}$$

i indicates the enterprise, j indicates the industry, k indicates the region, and t indicates the year. The explained variable ei represents the pollution emission intensity of enterprises, and the core explanatory variable epu represents the uncertainty of environmental policy. $X_{ijkt}$ is the control variable. $\lambda_{i}$ represents the firm fixed effect, $\lambda_{j}$ represents the industry fixed effect, $\lambda_{k}$ represents the region fixed effect, and $\lambda_{t}$ represents the year fixed effect. $\varepsilon_{ijkt}$ is the random perturbation term.

#### 2.3.2. Variable Selection

##### The Explained Variable Enterprise Pollution Emission Intensity

To measure the enterprise pollution emission intensity comprehensively and accurately, in this study, the comprehensive index method was used. Based on data availability and the harmfulness of pollutants, we mainly selected five individual indicators of industrial wastewater: chemical oxygen demand, industrial waste gas, sulfur dioxide, smoke, and dust emissions to measure the intensity of enterprise pollution emission [37]. First, the original data of these pollutant indicators are linearly standardized:

(25) $$rpol_{nit}=\frac{pol_{nit}-\min pol_{nit}}{\max pol_{nit}-\min pol_{nit}}$$

$pol_{nit}$ represents the emission of pollutant n from enterprise i in phase t. Max and min indicate the maximum and minimum emissions of pollutant n by all enterprises each year. Second, the adjustment coefficient of enterprise i pollutant n is calculated:

(26) $$w_{nit}=\frac{rpol_{nit}}{rpol_{nt}}$$

Then, $rpol_{nt}$ represents the average level of pollutant n discharged by all enterprises in China. Finally, by combining Equations (25) and (26), the comprehensive index of pollution emission of enterprise i can be obtained:

(27) eiit=16(∑nrpolnit×wnit)

The higher the value of ei, the greater the pollution emission intensity of the enterprise.

##### Core Explanatory Variable

The change in both external and internal environment increases the uncertainty of environmental policy, which causes a fluctuation in the enterprise’s sales revenue [38]. Therefore, the uncertainty of environmental policy can be measured by the fluctuation in the company’s sales income, and the standard difference in sales income can be used to measure the uncertainty of environmental policy [39]. Ghosh and Olsen [40] used the standard deviation of a company’s sales revenue to measure environmental policy uncertainty to eliminate the natural sales income’s growth.

The ordinary least squares method was used in this study to estimate the abnormal sales income of enterprises in the past three years.

(28) $$Sale=\varphi_{0}+\varphi_{1}Year+\varepsilon$$

Sale is the sales revenue of the enterprise and Year is the year. If the participation regression is the value of the past four years, then Year=1; if participation regression is the value of the past three years, then Year=2, and so on. The regression residual of Formula (28) is the abnormal sales revenue of the enterprise. The standard deviation of abnormal sales revenue for the past three years is calculated, and the standard deviation by is divided by the average sales revenue for the past three years to obtain the uncertainty of environmental policy without industry adjustment. The calculated environmental policy uncertainty is divided by the median of environmental policy uncertainty in the same industry to obtain the environmental policy uncertainty (epu).

##### Moderating Variables

The moderating variables are enterprise innovation, human capital, and foreign investment. Enterprise innovation (iq) is measured by the total number of patents granted per year; the existing literature suggests that employees without higher education are usually not considered within the scope of human capital [41]. However, the Chinese Industrial Enterprise Database only counted the educational level of employees in 2004, so the proportion of employees with college degrees in 2004 was used to measure the human capital of enterprises (rlzb). The amount of foreign investment in local enterprises (100 million) was selected to measure foreign investment (fi).

##### Other Variables

The selection of control variables mainly includes enterprise labor productivity (lp): to measure it, the total industrial value of the enterprise is divided by the total number of employees (10,000/person); wage level (gzsp): to measure it, enterprise wages and benefits are divided by the total number of employees (10,000/person); industrial structure (is): it is measured by the ratio of regional secondary industrial output value to total GDP; rationalization index (hlhzs): to measure it, the output value of each industry is divided by the corresponding number of people to obtain the per capita output value of each industry, and then the calculation results of each industry are added to obtain the rationalization index of each region; the dummy variable of state-owned enterprises (sfgy): 1 for absolute state-owned holding, 0 for other cases; import and export enterprise (ex): if the export delivery value of the enterprise is greater than 0, the ex value is 1; otherwise, 0; enterprise size (qygm): if the number of employees exceeds 1000, the value is 1 for large enterprises and 0 for small and medium enterprises; enterprise age (age): the enterprise age is calculated by subtracting the establishment year from the current year; capital labor ratio (zbld): to measure it, the total assets are divided by the number of workers (10,000/person); industry category dummy variable (hyfl): industry category 0 indicates polluting industry, and 1 indicates clean industry.

#### 2.3.3. Data Source and Processing

The data source of this study was the combined data of the China Industrial Enterprise Database, China Industrial Enterprise Pollution Emission Database, and China Patent Database. The China Industrial Enterprise Database contains the information of all state-owned enterprises and non-state-owned enterprises with an annual output value of more than RMB five million in China, mainly including enterprise name, organization code, legal representative, holding status, main business income, etc. The annual data volume is as high as 340,000 pieces, and the content is detailed. The statistical output value of enterprises accounts for more than 90% of the total output value of Chinese enterprises. The pollution emission database of China’s industrial enterprises is the most reliable data collected by the National Bureau of Statistics. It mainly collects statistics on enterprises with serious pollution discharge in each region. The table primarily includes the enterprise name, legal representative, organization code, wastewater, exhaust gas, and other pollution discharge indicators. For the purpose of research, this study merged the above two kinds of data. Firstly, the two kinds of data were processed according to the now commonly used processing methods [42]. Second, the company name and year were matched with patent data, and the matching data of the second and third steps were combined according to the organization code and year, and the duplicate values were removed. The combined data provided pollution emission information on enterprises with annual output values of more than RMB five million. Finally, certain enterprises with business status, state-owned status, enterprise size, industrial added value, or intermediate input missing, or negative values were deleted.

The State Intellectual Property Office is the authoritative source of China’s Patent Database, which mainly collects statistics on enterprises’ patent applications and authorization over the years. The content of China’s Patent Database mainly includes the enterprise name, organization code, invention patent, utility model patent, and design patent. The data of the Chinese Patent Database, Chinese Industrial Enterprise Database, and Chinese Industrial Enterprise Pollution Emission Database were combined using the above-discussed matching method. Finally, the combined data of the Chinese Patent Database, Chinese Industrial Enterprise Database, and China Industrial Enterprise Pollution Emission Database were obtained. About 510,000 enterprises participated in the empirical analysis.

As the latest statistical year of the China Industrial Enterprise Database is 2014, this study used statistical data from 2002 to 2014 to conduct empirical regression. Table 1 shows the descriptive statistics of variables in this study. There is a significant difference in pollution emission intensity among enterprises, with the minimum value being 0.001 and the maximum value being 18.570. There is also substantial difference in environmental policy uncertainty faced by enterprises, with the minimum value being 0.152 and the maximum value being 16.350.

## 3. Results

### 3.1. Basic Regression Results

Table 2 shows the results of stepwise regression. The uncertainty of environmental policy significantly aggravates the intensity of enterprise pollution emission. On the one hand, the increasing uncertainty of environmental policies aggravates the risks faced by enterprises, which then tend to keep more cash to cope with possible shocks [43]. Enterprise pollution investment needs to introduce a large amount of advanced purification equipment and environmental protection talents, but emission investment generally has the characteristics of low early return and high risk. This further dissuades enterprises from upgrading production emission technology and introducing human capital. On the other hand, the increasing uncertainty of environmental policies makes it difficult for foreign investors to accurately predict their investment returns. The operating and financial risks of enterprises increase significantly, leading to the reduction in foreign investment and the external financing ability of enterprises, and lowering the investment of enterprises in sewage discharge equipment. Finally, the increase in environmental policy uncertainty significantly intensifies the pollution emission intensity of enterprises.

### 3.2. Heterogeneity Analysis

#### 3.2.1. Enterprise Export Status Heterogeneity

We divided enterprises into exporting and non-exporting enterprises and investigated whether the impact of environmental policy uncertainty on enterprise pollution emissions is heterogeneous because of the different export statuses of enterprises. As can be seen from Columns (1) and (2) of Table 3, the environmental policy uncertainty of both export and non-export enterprises has a significant positive impact on enterprise pollution emission intensity; however, the influence coefficient of non-export enterprises is greater than that of export enterprises. On the one hand, export companies are forced to improve their pollution techniques because their products are subject to stricter inspections abroad. On the other hand, through “learning in export,” export enterprises obtain more advanced sewage discharge technology and experience from abroad, which reduces the pollution emission intensity of export enterprises. Therefore, environmental policy uncertainty has little influence on the pollution emission intensity of export enterprises. However, the relatively backward pollution discharge technology of non-export enterprises leads to higher pollution discharge cost, and the pollution discharge of enterprises is significantly affected by environmental policies. Therefore, when the uncertainty of environmental policy increases, it impacts the pollution emission of non-export enterprises.

#### 3.2.2. Heterogeneity of Firm Size

Enterprises can be categorized as large- and small- and medium-sized enterprises based on whether the number of employees exceeds 1000 according to the classification standards of “Measures for The Classification of Statistically Large, Small and Medium-sized Enterprises (2017).” We aimed to investigate whether the impact of environmental policy uncertainty on enterprise pollution emissions is heterogeneous because of different enterprise sizes. Columns (3) and (4) of Table 3 show that environmental policy uncertainty has a greater impact on the pollution emission intensity of large enterprises. The pollution emission intensity of large enterprises is generally higher than that of small- and medium-sized enterprises. When the uncertainty of environmental policy increases, large enterprises reduce their investment in emission reduction technologies and reserve more cash to cope with possible shocks, because of which, their pollution emission increases. Therefore, the pollution emission of large enterprises is greatly affected by the uncertainty of environmental policy. Alternatively, small- and medium-sized enterprises have less pollution emissions, and the increase in environmental policy uncertainty has less impact on their pollution emissions.

#### 3.2.3. Enterprise’s Age Heterogeneity

Based on the median time of establishment, enterprises are divided into young and old enterprises. Columns (1) and (2) of Table 4 show that environmental policy uncertainty has a greater impact on the pollution emission intensity of young enterprises compared to old enterprises. The establishment period of young enterprises is relatively short, and they lack advanced sewage discharge equipment, abundant capital, and experience in dealing with environmental policy uncertainty. Alternatively, old enterprises have relatively better sewage discharge equipment, abundant sewage discharge capital, and greater experience in dealing with environmental policy uncertainty. So, the impact of environmental policy uncertainty on their pollution emission is less compared to that on young enterprises.

#### 3.2.4. Enterprise’s Type Heterogeneity

Based on the median capital labor ratio of enterprises, they are categorized as labor-intensive and capital-intensive enterprises. Columns (3) and (4) of Table 4 show that environmental policy uncertainty has a significantly positive impact on enterprise pollution emission intensity in the case of capital-intensive enterprises, but not in the case of labor-intensive enterprises, in which environmental policy uncertainty has no significant impact on enterprise pollution emission intensity. As labor-intensive enterprises are mostly light industrial enterprises, their pollution emission intensity is relatively low. Alternatively, capital-intensive enterprises, which are mostly heavy industrial enterprises, have higher pollution emission intensity and are significantly affected by the uncertainty of environmental policy. Therefore, when environmental policy uncertainty changes, it has a greater impact on the pollution emission intensity of capital-intensive enterprises than labor-intensive enterprises.

#### 3.2.5. Enterprise’s Ownership Heterogeneity

Enterprises are divided into state-owned and non-state-owned enterprises depending on whether they are state holding enterprises or not. Columns (1) and (2) of Table 5 show that the uncertainty of environmental policy has a greater impact on the pollution emission intensity of state-owned enterprises, but a smaller impact on that of non-state-owned enterprises. As profits or emission reduction in state-owned enterprises are mostly affected by national policies, when the uncertainty of environmental policy changes, it has a significant impact on the pollution emission intensity of state-owned enterprises. Alternatively, non-state-owned enterprises are owned by private enterprises and foreign-funded enterprises, and their pollution emission intensity is less affected by environmental policy uncertainty.

#### 3.2.6. Industry Category Heterogeneity

Companies are divided into clean and polluting enterprises according to their industry. Columns (3) and (4) of Table 5 show that the uncertainty of environmental policy has a smaller impact on the pollution emission intensity of clean enterprises compared to polluting enterprises. As most clean enterprises are light industry and handicraft industry enterprises, their environmental pollution emission intensity is low; so, when the uncertainty of environmental policy increases, this has an insignificant impact on the pollution emission intensity of clean industry enterprises. However, the pollution emission intensity of enterprises in polluting industries is high. When the government’s environmental regulation intensity is low, the pollution emission of enterprises is high, and when the government’s environmental regulation intensity is high, enterprises reduce their pollution emission. Therefore, when the uncertainty of environmental policy increases, it has a greater impact on the pollution emission intensity of enterprises in polluting industries.

#### 3.2.7. Region heterogeneity

China is divided into eastern, central, and western regions to study whether the environmental policy uncertainty in different regions has different effects on enterprises’ pollution emission intensity. The results are shown in Table 6. Due to the better economic foundation in the eastern region, the government will force enterprises to update pollution equipment when the intensity of environmental regulation increases. Then, enterprises recruit a large number of scientific and technological innovative talents, so the enterprises’ pollution emission technology in the eastern region is more advanced. At the same time, some polluting enterprises in the eastern region choose to migrate to the neighboring central region due to the increase in emission costs, so the enterprises’ pollution emission intensity in the central region is more serious. When environmental uncertainty is increased due to the policy change, its impact on the enterprises’ pollution emission intensity in the eastern region is low. On the one hand, the central region undertakes the transfer of polluting enterprises in the eastern region, so the enterprises’ pollution emission intensity in the central region is higher. When the government’s environmental regulation intensity is low, the pollution emission intensity increases. When the government’s environmental regulation intensity increases, enterprises have to reduce the pollution emission intensity. Therefore, the change in environmental policy uncertainty in the central region has a greater impact on the pollution emission intensity. The development of the western region is relatively backward, and the economy mainly depends on resource extraction enterprises, which leads to high pollution emission intensity. Due to the inherent production nature and enterprises’ development environment, it is difficult to reduce the pollution emission intensity in the western region. Therefore, when environment policy uncertainty increases, the change in pollution emission intensity in the western region is still low.

### 3.3. Robustness Test

#### 3.3.1. Transform Core Explanatory Variables

In this section, the uncertainty of environmental policy is re-measured to verify whether its impact on enterprise pollution emission intensity is robust. The previous article mainly refers to the Ghosh and Olsen [40] algorithm. Environmental policy uncertainty is calculated based on abnormal sales income and standard deviation in the past three years. In this section, three years of calculation are raised to five years, and the same method is used to measure environmental policy uncertainty (epu1). Stepwise regression results are shown in Table 7. It can be seen that the environmental policy uncertainty still has a significant positive effect on the pollution emission intensity of enterprises, indicating that this study’s conclusions are robust and reliable.

#### 3.3.2. Transform the Interpreted Variable

The pollution emission intensity of enterprises mentioned above was measured using the comprehensive index method. In this section, five single pollution emissions of enterprises are used to measure the pollution emission intensity of enterprises. The regression results are shown in Table 8. It can be seen that environmental policy uncertainty still significantly promotes the improvement of the pollution emission intensity of enterprises, indicating that the conclusions of this study are robust and reliable.

#### 3.3.3. Eliminate the Sample of Entering and Exiting Enterprises

The entry and exit of enterprises in the sample period interferes with the impact of environmental policy uncertainty on enterprise pollution emissions. Therefore, this section deletes entering and exiting enterprises in the sample period for robustness tests. The regression results are shown in Column (1) of Table 9. The influence coefficient of environmental policy uncertainty on enterprise pollution emission intensity is significantly positive, indicating that the research conclusions in this study are robust and reliable.

#### 3.3.4. Change Sample Period and Delete the Time Dummy Variable

In this study, macro-variables were controlled to eliminate the influence of time factor on empirical results, but time factors may still have had an impact on the results. Robustness was analyzed from two perspectives. (1) The sample period of this study was 2002–2014. The State Environmental Protection Administration set up six inspection centers in 2006. The Environmental Protection Law was introduced in 2008, and ecological progress was proposed at the 18th National Congress of the Communist Party of China in 2012. To control the influence of these factors, only samples from 2012 to 2014 are used for regression in this section. (2) We considered deleting the time dummy variable and substituting the rest of the dummy variables into the regression model to control the influence of the time effect. The results are shown in Columns (2) and (3) of Table 9. The influence coefficient of environmental policy uncertainty on enterprise pollution emission intensity is significantly positive, indicating that the conclusions of this study are robust and reliable.

#### 3.3.5. Deleting Outliers

To eliminate the influence of outliers on regression results, variables were treated with Winsor at the level of 1%. The regression results are shown in Column (4) of Table 9. The influence coefficient of environmental policy uncertainty on enterprise pollution emission intensity is significantly positive, indicating that the research conclusions are robust and reliable. 

### 3.4. Endogeneity Test

As environmental policy uncertainty is caused by the fluctuation of national macro-policy, and the micro-behavior of individual enterprises can hardly affect the macro-environmental policy, there is almost no reverse causality between enterprise pollution emissions and environmental policy uncertainty. Additionally, the fixed effects of enterprises, industries, regions, and years are strictly controlled in the empirical analysis of this study, which effectively avoids the endogenous problems caused by omitted variables. As eliminating the lag term of enterprise pollution emission intensity may affect the results, referring to Fang et al. (2015), this study added the variable L.ei into the regression Formula [43]; the results are shown in Column (5) of Table 9. The significance of the explanatory variables remains unchanged.

### 3.5. Mechanism Analysis

Based on the above theoretical analysis, environmental policy uncertainty can affect the pollution emission intensity of enterprises in three ways: enterprise innovation, human capital, and foreign investment. This section empirically tests the impact of environmental policy uncertainty on enterprise pollution emission intensity through these three channels. The specific model is as follows:

(29) $$ei_{ijkt}=\beta_{0}+\beta_{1}epu_{ijkt}+\beta_{2}iq_{ijkt}+\beta_{3}epu_{ijkt}\cdot iq_{ijkt}+\beta_{4}X_{ijkt}+\lambda_{i}+\lambda_{j}+\lambda_{k}+\lambda_{t}+\varepsilon_{ijkt}$$

(30) $$ei_{ijkt}=\beta_{0}+\beta_{1}epu_{ijkt}+\beta_{2}rlzb_{ijkt}+\beta_{3}epu_{ijkt}\cdot rlzb_{ijkt}+\beta_{4}X_{ijkt}+\lambda_{i}+\lambda_{j}+\lambda_{k}+\lambda_{t}+\varepsilon_{ijkt}$$

(31) $$ei_{ijkt}=\beta_{0}+\beta_{1}epu_{ijkt}+\beta_{2}fi_{ijkt}+\beta_{3}epu_{ijkt}\cdot fi_{ijkt}+\beta_{4}X_{ijkt}+\lambda_{i}+\lambda_{j}+\lambda_{k}+\lambda_{t}+\varepsilon_{ijkt}$$

Formula (29) shows that environmental policy uncertainty affects enterprise pollution emission intensity through firm innovation, Formula (30) shows that environmental policy uncertainty affects enterprise pollution emission intensity through human capital, and Formula (31) shows that environmental policy uncertainty affects enterprise pollution emission intensity through foreign investment. The regression results are shown in Table 10.

#### 3.5.1. Enterprise Innovation

The purpose of enterprise investment is profit maximization. With an increase in environmental policy uncertainty, the silent risk of enterprise investment and innovation also increases. Moreover, innovation investment is a project with high risk and a long cycle, so enterprise innovation investment needs sufficient capital guarantee. When the uncertainty of the environmental policy facing enterprises increases, the uncertainty of enterprise innovation investment income increases, and to reduce risks, enterprise managers decrease investment in innovation. This lowers the innovation capacity of enterprises. As can be seen from Column (1) of Table 10, the interaction coefficient between environmental policy uncertainty and enterprise innovation is significantly negative. It reveals that environmental policy uncertainty aggravates enterprises’ pollution emission by inhibiting the improvement of enterprises’ innovation ability. Thus, Hypothesis 1 is verified.

#### 3.5.2. Human Capital

According to the classical financial theory, when an enterprise faces greater uncertainty in the external environment, it reserves more cash to cope with unknown shocks [44]. When the uncertainty of environmental policy increases, enterprises expect that financing in the capital market will be full of difficulties, the human capital input of enterprises has the characteristics of longer term and less obvious income, and the adjustment cost is lower than other factors of production. Therefore, enterprises decelerate the introduction of human capital to retain more cash to cope with unknown impacts of environmental policy uncertainty, which reduces the human capital stock of enterprises. Additionally, when the environmental uncertainty of enterprises increases, enterprises tend to reduce their operating costs in order to retain more cash to face possible future shocks. It is common for enterprises to lay off employees, increase the labor supply time of employees, and reduce salaries. Employees are faced with reduced income and increased labor intensity, which will further increase the incidence of disease, thus damaging the healthy human capital of employees. Column (2) of Table 10 shows that the interaction coefficient of environmental policy uncertainty and human capital is significantly negative, indicating that environmental policy uncertainty aggravates enterprise pollution emissions by inhibiting the increase in enterprise human capital stock. Thus, Hypothesis 2 is verified.

#### 3.5.3. Foreign Investment

The change in external environment is an important factor affecting foreign investment, the main purpose of which is profit. When the uncertainty of investors’ profit expectation increases, the opportunity cost of enterprise investment also increases, and foreign capital flows to projects with more definite investment returns. Therefore, the increased uncertainty of environmental policy reduces foreign investment expectations. Column (3) of Table 10 shows that the interaction coefficient between environmental policy uncertainty and foreign investment is significantly negative, indicating that environmental policy uncertainty aggravates enterprises’ pollution emissions by inhibiting foreign investment. Thus, Hypothesis 3 is verified.

## 4. Discussion

Based on the combined data of the China Industrial Enterprise Database, China Industrial Enterprise Pollution Emission Database, and China Patent Database from 2002 to 2014, we empirically analyzed the impact of environmental policy uncertainty on enterprise pollution emission.

### 4.1. Contribution

The marginal contributions of this study are as follows: First, at the research level, the existing literature mainly discusses the impact of environmental regulations on regional environmental pollution emissions from a macro-perspective; the micro-viewpoint is the basis and key to understand the macro-perspective. From the perspective of micro-enterprises, this study was mainly based on the Chinese Industrial Enterprise Pollution Emission Database, and chemical oxygen demand, industrial waste gas, sulfur dioxide, smoke, and dust emissions were selected to measure the intensity of enterprise pollution emission. The composite index method makes the measurement result closer to the real enterprise pollution emission intensity. It also systematically discusses the influence of environmental policy uncertainty on enterprise pollution emissions to further refine the existing research and establishes the micro-basic cognition of the relationship between environmental policy uncertainty and enterprise pollution emissions. Thus, it makes up for the deficiencies of existing research in the field of micro-enterprises.

Second, in terms of research content and perspectives: (1) from the perspective of increasing environmental policy uncertainty, this article discussed the impact and mechanism of environmental policy uncertainty on enterprise pollution emissions and provides a new research perspective for enterprise pollution emissions; and (2) there is often summing bias in regional summing data, which may cover up or change the real relationship between variables in empirical tests, and as macro-research ignores the heterogeneity of enterprises, it is difficult to investigate how enterprises’ emission reduction behaviors with different characteristics respond to environmental policy uncertainties. This article discussed the heterogeneity of enterprise export status, enterprise size, enterprise age, enterprise type, enterprise ownership, and enterprise industry category, which helps to form a comprehensive understanding of the relationship between environmental policy uncertainty and enterprise pollution emissions.

Third, regarding the mechanism of action, the existing literature on the mechanism of enterprise emission reduction is increasingly mature. However, from a new perspective of environmental policy uncertainty and micro level, the effectiveness of these mechanisms is questionable. Based on the theoretical model, this study revealed that environmental policy uncertainty affects enterprise pollution emissions through three channels: technological innovation, human capital, and foreign investment. It further deepened the understanding of the complete logical chain of environmental policy uncertainty to enterprise pollution discharge.

Forth, ordinary least squares was selected as the analysis method, and the interference of time, industry, region, and individual was controlled, so that the correlation coefficient between environmental policy uncertainty and enterprise pollution emission intensity was closer to the reality.

### 4.2. Limitations

The research still has certain limitations and should be further deepened. First, in view of the limitations of research methods, this paper selected panel data to analyze the heterogeneity of the Enterprise Pollution Emission Intensity and influencing factors in China; however, it lacked an analysis of the dynamic evolution of space. In the future, appropriate research methods will be selected to conduct a more in-depth spatial dynamic analysis of the Enterprise Pollution Emission Intensity. Second, since the latest data were only collected in 2014, if there are updated data in the future, we will further update the data in the following study.

## 5. Conclusions

Based on the combined data of the China Industrial Enterprise Database, China Industrial Enterprise Pollution Emission Database, and China Patent Database from 2002 to 2014, we empirically analyzed the impact of environmental policy uncertainty on enterprise pollution emission. The results show that:

First, the impact of environmental policy uncertainty on enterprise emission reduction was discussed theoretically and empirically. Second, the heterogeneity of environmental policy uncertainty on enterprise pollution emission was discussed from the perspectives of enterprise export status, enterprise size, enterprise age, enterprise type, enterprise ownership, enterprise industry category, and different region. Finally, we empirically examined whether environmental policy uncertainty can increase enterprise pollution emissions by inhibiting enterprise innovation, human capital, and foreign investment.

The conclusions of this study are as follows: (1) The uncertainty of environmental policy significantly intensifies the pollution emission intensity of enterprises and aggravates regional environmental pollution. (2) Environmental policy uncertainty intensifies enterprises’ pollution emission intensity through three transmission channels: enterprise innovation, human capital, and foreign investment. In other words, environmental policy uncertainty intensifies enterprises’ pollution emission intensity by inhibiting the improvement of their innovation ability, reducing their human capital stock, and discouraging foreign investment. (3) Environmental policy uncertainty has significant heterogeneity on enterprise pollution emissions, that is, environmental policy uncertainty has a greater impact on non-export enterprises, large enterprises, young enterprises, capital-intensive enterprises, state-owned enterprises, and enterprises in polluting industries and central regions.

### Policy Enlightenment

The conclusions of this study have enlightening significance for the adjustment of environmental policy and enterprise green transformation. (1) The government should give full play to the decisive role of the market in resource allocation, strengthen macro-regulation, and reduce uncertainties in environmental policies. Currently, China needs enormous external capital, new talent, and green technology to promote economic transformation and upgradation. The government should build a stable economic environment to help enterprises acquire foreign capital, advanced technology, and new talents, and accelerate the transformation or elimination of high-pollution, high-consumption enterprises. (2) The government should construct a stable and efficient compound environmental regulation policy system. Although the government’s environmental regulation measures can curb pollution emission by enterprises, the frequent introduction of environmental policies increases environmental policy uncertainty, which inhibits enterprise innovation, human capital, and foreign investment. Therefore, the government should maintain the sustainability of local environmental regulation policies, effectively promote the coordination and cooperation of heterogeneous environmental regulation tools, and reduce the negative impact of environmental policy uncertainty on enterprise emission reduction. (3) In the process of policy formulation and implementation, the government needs to further refine specific measures. Due to the significant heterogeneity of environmental policy uncertainty on enterprise pollution emission, the government needs to formulate detailed measures. As such, we will focus on monitoring pollution emissions of non-export enterprises and small enterprises, so as to minimize the negative impact of environmental policy uncertainty on enterprise emission reduction. In general, this study introduced a new research perspective and microscopic evidence for the green transformation development of Chinese enterprises.

## Figures and Tables

**Table 1:** Descriptive statistics of variables.

Variables	Std. Dev	Mean	Min	Max
ei	1.495	0.254	0.001	18.570
epu	1.877	1.589	0.152	16.350
lp	0.256	0.011	0.001	90.340
gzsp	1.002	0.048	0.001	261.200
is	0.220	0.816	0.494	3.662
hlhzs	0.139	0.216	0.017	0.932
iq	9.310	0.002	0.001	3093
rlzb	0.053	0.030	0.001	0.277
fi	0.173	0.155	0.001	0.718

**Table 2:** Stepwise regression results.

Variables	(1)	(2)	(3)	(4)	(5)
	ei	ei	ei	ei	ei
epu	0.017 ***	0.017 ***	0.017 ***	0.017 ***	0.017 ***
	(0.002)	(0.002)	(0.002)	(0.002)	(0.002)
lp		0.235 ***	0.302 ***	0.302 ***	0.302 ***
		(0.016)	(0.025)	(0.025)	(0.025)
gzsp			−0.017 ***	−0.017 ***	−0.017 ***
			(0.005)	(0.005)	(0.005)
is				0.191 ***	0.113 ***
				(0.029)	(0.031)
hlhzs					−0.615 ***
					(0.089)
Enterprise	Yes	Yes	Yes	Yes	Yes
Industry	Yes	Yes	Yes	Yes	Yes
Time	Yes	Yes	Yes	Yes	Yes
Reigon	Yes	Yes	Yes	Yes	Yes
Obs	204,355	204,318	200,585	200,585	200,585
R-squared	0.040	0.041	0.042	0.042	0.042

Note: *** denote the significance at 1% levels.

**Table 3:** Results of enterprise export status and size heterogeneity.

Variables	(1)	(2)	(3)	(4)
	Export Enterprises	Non-Export Enterprise	Big Companies	Small- and Medium-Sized Enterprises
	ei	ei	ei	ei
epu	0.013 ***	0.017 ***	0.039 ***	0.003 **
	(0.003)	(0.002)	(0.009)	(0.001)
Control variables	Yes	Yes	Yes	Yes
Enterprise	Yes	Yes	Yes	Yes
Industry	Yes	Yes	Yes	Yes
Time	Yes	Yes	Yes	Yes
Reigon	Yes	Yes	Yes	Yes
Obs	60,104	140,481	28,918	171,667
R-squared	0.020	0.051	0.115	0.038

Note: **, *** denote the significance at 5%, 1% levels, respectively.

**Table 4:** Results of enterprise age and type heterogeneity.

Variables	(1)	(2)	(3)	(4)
	Younger Enterprises	Older Enterprises	Labor-Intensive Enterprises	Capital-Intensive Enterprises
	ei	ei	ei	ei
epu	0.018 ***	0.015 ***	0.002	0.031 ***
	(0.003)	(0.002)	(0.002)	(0.003)
Control variables	Yes	Yes	Yes	Yes
Enterprise	Yes	Yes	Yes	Yes
Industry	Yes	Yes	Yes	Yes
Time	Yes	Yes	Yes	Yes
Reigon	Yes	Yes	Yes	Yes
Obs	87,389	126,015	99,995	100,590
R-squared	0.026	0.058	0.031	0.048

Note: *** denote the significance at 1% levels.

**Table 5:** Results of enterprise ownership and industry category heterogeneity.

Variables	(1)	(2)	(3)	(4)
	State-Owned Enterprises	Non-State-Owned Enterprises	Clean Industry	Polluting Industries
	ei	ei	ei	ei
epu	0.044 ***	0.004 ***	0.016 ***	0.018 ***
	(0.007)	(0.002)	(0.005)	(0.002)
Control variables	Yes	Yes	Yes	Yes
Enterprise	Yes	Yes	Yes	Yes
Industry	Yes	Yes	Yes	Yes
Time	Yes	Yes	Yes	Yes
Reigon	Yes	Yes	Yes	Yes
Obs	35,504	165,079	36,518	164,067
R-squared	0.069	0.023	0.123	0.022

Note: *** denote the significance at 1% levels.

**Table 6:** Results of enterprise ownership and industry category heterogeneity.

Variables	(1)	(2)	(3)
	Eastern Region	Central Region	Western Region
	ei	ei	ei
epu	0.013 ***	0.031 ***	0.015 ***
	(0.002)	(0.006)	(0.007)
Control variables	Yes	Yes	Yes
Enterprise	Yes	Yes	Yes
Industry	Yes	Yes	Yes
Time	Yes	Yes	Yes
Reigon	Yes	Yes	Yes
Obs	127,373	38,521	34,691
R-squared	0.032	0.044	0.036

Note: *** denote the significance at 1% levels.

**Table 7:** Change the core explanatory variable—environmental policy uncertainty.

Variables	(1)	(2)	(3)	(4)	(5)
	ei	ei	ei	ei	ei
epu1	0.014 *	0.015 *	0.015 *	0.014 *	0.015 *
	(0.008)	(0.008)	(0.008)	(0.008)	(0.008)
lp		0.343 ***	0.316 ***	0.316 ***	0.315 ***
		(0.035)	(0.047)	(0.047)	(0.047)
gzsp			0.013	0.013	0.013
			(0.015)	(0.015)	(0.015)
is				0.152 ***	0.032
				(0.059)	(0.063)
hlhzs					−1.221 ***
					(0.226)
Enterprise	Yes	Yes	Yes	Yes	Yes
Industry	Yes	Yes	Yes	Yes	Yes
Time	Yes	Yes	Yes	Yes	Yes
Reigon	Yes	Yes	Yes	Yes	Yes
Obs	64,776	64,763	62,785	62,785	62,785
R-squared	0.038	0.040	0.041	0.041	0.041

Note: *, *** denote the significance at 10%, 1% levels, respectively.

**Table 8:** Change the explained variable—pollution emission intensity of enterprises.

Variables	(1)	(2)	(3)	(4)	(5)
	Discharge of Industrial Wastewater	Chemical Oxygen Demand Emissions	Total Industrial Exhaust Emissions	Sulfur Dioxide Emissions	Smoke and Dust Emission
epu	1.312 ***	0.370 **	0.934 ***	1.188 ***	5.889 ***
	(0.337)	(0.180)	(0.172)	(0.204)	(1.713)
Control variables	Yes	Yes	Yes	Yes	Yes
Enterprise	Yes	Yes	Yes	Yes	Yes
Industry	Yes	Yes	Yes	Yes	Yes
Time	Yes	Yes	Yes	Yes	Yes
Reigon	Yes	Yes	Yes	Yes	Yes
Obs	177,652	192,764	82,159	191,119	147,529
R-squared	0.005	0.016	0.108	0.066	0.037

Note: **, *** denote the significance at 5%, 1% levels, respectively.

**Table 9:** Regression results of eliminate the entry and exit samples, adjust the sample period and Winsor.

Variables	(1)	(2)	(3)	(4)	(5)
	Eliminate Entry Exit Sample	2012–2014	Delete the Time Dummy Variable	Winsor	Add Variable L.ei
	ei	ei	ei	ei	ei
epu	0.008 ***	0.046 ***	0.017 ***	0.018 ***	0.018 ***
	(0.002)	0.004	(0.002)	(0.002)	(0.005)
Control variables	Yes	Yes	Yes	Yes	Yes
Enterprise	Yes	Yes	Yes	Yes	Yes
Industry	Yes	Yes	Yes	Yes	Yes
Time	Yes	Yes	Yes	Yes	Yes
Region	Yes	Yes	Yes	Yes	Yes
Obs	172,969	65,163	200,585	200,585	32,153
R-squared	0.048	0.031	0.042	0.047	0.039

Note: *** denote the significance at 1% levels.

**Table 10:** Mechanism test results.

Variables	(1)	(2)	(3)
	ei	ei	ei
epu	0.017 ***	−0.002	0.021 ***
	(0.002)	(0.032)	(0.003)
iq	0.172 ***		
	(0.035)		
epu·iq	−0.017 *		
	(0.009)		
rlzb		5.088 ***	
		(1.445)	
epu·rlzb		−1.826 **	
		(0.710)	
fi			−0.248 ***
			(0.067)
epu·fi			−0.021 **
			(0.010)
Control variables	Yes	Yes	Yes
Enterprise	Yes	Yes	Yes
Industry	Yes	Yes	Yes
Time	Yes	Yes	Yes
Reigon	Yes	Yes	Yes
Obs	200,585	622	200,585
R-squared	0.043	0.140	0.043

Note: *, **, *** denote the significance at 10%, 5%, 1% levels, respectively.

## Data Availability

The Ministry of Ecology and Environment of the People’s Republic of China (https://www.mee.gov.cn/, accessed on 15 March 2022); the National Bureau of Statistics (http://www.stats.gov.cn/, accessed on 16 March 2022); China National Intellectual Property Administration (https://www.cnipa.gov.cn/, accessed on 16 March 2022); China Patent Information Center (http://www.cnpat.com.cn, accessed on 17 March 2022). EPSDATA (https://www.epsnet.com.cn/index.html#/, Home, accessed on 17 March 2022).
